# Exploring spatial variations in level and predictors of unskilled birth attendant delivery in Bangladesh using spatial analysis techniques: Findings from nationally representative survey data

**DOI:** 10.1371/journal.pone.0275951

**Published:** 2022-10-25

**Authors:** Md. Rahman Mahfuzur, Md. Arif Billah, Nicola Liebergreen, Manoj Kumer Ghosh, Md. Shafiul Alam, Md. Armanul Haque, Abdullah Al-Maruf

**Affiliations:** 1 Department of Population Science and Human Resource Development, Sir Jagadish Chandra Bose Academic Building, University of Rajshahi, Rajshahi, Bangladesh; 2 Department of Social Work and Counselling, Faculty of Business, Economics and Social Development, Universiti Malaysia Terengganu, Kuala Nerus, Terengganu, Malaysia; 3 Bioethics Centre, University of Otago, Dunedin, New Zealand; 4 Department of Geography and Environmental Studies, University of Rajshahi, Rajshahi, Bangladesh; 5 Information Science & Library Management, University of Rajshahi, Rajshahi, Bangladesh; University of North Carolina at Chapel Hill Gillings School of Global Public Health, UNITED STATES

## Abstract

**Background:**

Bangladesh has failed to meet the United Nations goal for reducing maternal mortality in the last decade. The high prevalence of unskilled birth attendant (UBA) delivery (47%) has resulted in negative consequences for the health of mothers and newborn babies in the country. Spatial variations in UBA delivery and its predictors are yet to be explored in Bangladesh, which could be very helpful in formulating cost-effective policies for reducing that. This study examines the spatial variations in UBA delivery and its predictors in Bangladesh.

**Methods:**

This study analyzed the characteristics of 672 clusters extracted from the 2017/18 Bangladesh Demographic and Health Survey, and healthcare facility data from the 2017 Bangladesh Health Facility Survey. These data were analyzed using descriptive and spatial analyses (hot spot analysis, Ordinary Least Squares Regression, and Geographically Weighted Regression) techniques.

**Results:**

Statistically significant hot spots of UBA delivery were concentrated in parts of the Mymensingh, Sylhet, Barishal, and Rangpur regions, while Khulna was the safest region. Predictive strengths of the statistically significant predictors of spatial variation in UBA delivery were observed to vary considerably across the regions. Poorest household wealth status and less than four antenatal care contacts emerged as strong predictors of UBA delivery in all the aforementioned hot spot-stricken regions, except Barisal. Additionally, primiparity and all secondary education or higher were strong predictors of lower UBA delivery rates in Mymensingh and Sylhet, while poorer household wealth status was also a strong predictor of UBA delivery in Sylhet. Multiparity was an additional strong predictor of UBA delivery in Rangpur. In Barisal, only poorer household wealth status exerted a strong positive influence on UBA delivery.

**Conclusions:**

The remarkable spatial variations in UBA delivery and its predictors’ strengths indicate that geographically-targeted interventions could be a cost-effective method for reducing the UBA delivery prevalence in Bangladesh, thereby improve maternal and child health.

## Background

In the year 2000, The United Nations (UN) Millennium Declaration set eight Millennium Development Goals (MDGs) for its member states to reach by 2015. One of these goals was to cut the maternal mortality ratio (MMR) by 75% (MDG-5). MMR refers to maternal deaths per 100,000 live births. After failing to achieve this reduction in the MMR by 2015, the global community reviewed the goals and redeveloped them as 17 Sustainable Development Goals (SDGs). One of these SDGs is to cut MMR globally to less than 70 by 2030. Approximately 99% of all maternal deaths globally occur in developing regions, with South Asia experiencing about 22% and Sub-Saharan Africa experiencing 66% of global maternal deaths [1].

Bangladesh is a developing country in South Asia. Although the estimates of MMR in Bangladesh obtained by different agencies and organizations vary, all estimates are very high. Despite experiencing a remarkable decline in maternal and child mortality during the 2000s, the country has recently struggled to achieve the goals relating to maternal mortality [2]. According to the Bangladesh Maternal Mortality and Health Care Survey (BMMS), the MMR in Bangladesh declined from 322 in 2001, to 194 in 2010 [3, 4], and the government aimed to achieve MDG-5 by further reducing the MMR to 143 by 2015 [5]. Despite this commitment, the MMR in Bangladesh almost stalled during the 2010–2016 period, with the estimated rate sitting at 196 (95% CI: 159–234) in 2016; a very similar rate to the 2010 MMR estimate of 194 (95% CI: 153–236) [3, 4]. According to the Bangladesh Bureau of Statistics and the World Bank, the MMRs in Bangladesh in 2017 were, respectively, 178 and 173, which were within the margin of error of the 2016 estimate provided by BMMS [4, 6]. According to the data published by the World Bank, the MMR in Bangladesh in 2017 was remarkably higher than the average MMR in South Asia (163), and that of all other South Asian countries, other than Nepal. The MMRs in Sri Lanka, the Maldives, Pakistan, India and Nepal in 2017 were, respectively, 36, 53, 140, 145 and 186 [6].

One of the factors directly contributing to the high MMR in Bangladesh is the high prevalence of deliveries that are assisted by unskilled birth attendants (UBAs) only [7]. UBAs are often older women with no formal midwifery education and training. These women have learnt their midwifery skills through their own childbirth experiences and working with other UBAs and have no medical training. As a result, some unsafe practices occur, including poor hygiene practices and delays in referring cases of excessive postpartum bleeding to trained personnel [8]. In contrast to a UBA, a skilled birth attendant (SBA) is “an accredited health professional–such as a midwife, doctor or nurse–who has been educated and trained to proficiency in the skills needed to manage normal (i.e. uncomplicated) pregnancies, childbirth and the immediate postnatal period, and in the identification, management and referral of women and neonates for complications” [9]. The assistance of a SBA is very important for the health of both the mother and the newly born child, as the majority of maternal and newborn deaths occur during childbirth, or very soon after [10–12]. Every woman–rich or poor–has a 15% risk for complications around the delivery time [9], but an SBA can significantly reduce the risk of delivery-related complications by detecting and managing the issues, or by referring patients elsewhere if complications arise [13].

Despite the life-saving benefits of using SBAs, a substantial proportion of births globally (16%) took place with the assistance of UBAs only during the 2015–2021 period. During this period, the rate of UBA delivery was very low in Northern America and Europe (1%), but was very high in Sub-Saharan Africa (36%) and South Asia (18%) [14]. Bangladesh had the highest UBA delivery rate in South Asia. In 2017, about 47% of all deliveries in Bangladesh were assisted by UBAs [15]. In comparison, the UBA delivery rates in other South Asian countries, such as the Maldives (in 2016), India (in 2015), Pakistan (in 2017), and Nepal (in 2016) were, respectively, 0.5%, 17%, 27% and 37%. After a substantial improvement in the delivery care services, the UBA delivery rate in India was reduced to 9.5% in 2020 [16], which suggests that the same may be possible in Bangladesh.

Most existing studies of the determinants of UBA use have analyzed the UBA use in African countries, including Ethiopia, Kenya, Nigeria, Senegal, South Sudan and Tanzania. At the aggregate level, these studies show that women with the following attributes are less likely to use UBAs: higher age (35–49 years), secondary or higher education of mothers and husbands/partners, higher wealth status, job possession by mothers, exposure to mass media, urban residency, four or more (i.e. at least four) antenatal care (ANC) contacts (or visits), one or more ANC contacts, multiparity, knowledge of pregnancy complication(s), and having a short travelling time to a maternity center [17–26]. Some articles that studied the use of SBA delivery in Bangladesh showed that higher age at first birth, secondary or higher education of mothers and husbands, higher wealth status, job possession by mothers, urban residence, caesarean delivery, having any ANC by an SBA, two or more ANC visits, 0–1 parity, and complications during pregnancy were significantly associated with a higher rate of SBA use during delivery [27–29]. In contrast to the aforementioned findings, Bhowmik et al. (2019) found that, in Bangladesh, women who were working and participated in household decision-making were less likely to use an SBA during their delivery [29].

There is also literature that relates to studies in Ethiopia and Ghana that performed spatial analysis for examining the use of UBAs for deliveries. These studies found spatial variations in the level and strengths of the predictors of UBA delivery [30–32]. These studies showed that women’s literacy, wealth status, income level, access to health insurance, media exposure, and perception of their distance from a health facility as not a big problem were negatively correlated with UBA use. Community literacy and higher community wealth status also exerted negative effects on the use of UBAs during delivery.

As the variation in the utilization of UBAs and its predictors may depend upon the variation in the characteristics of comparatively smaller geographic areas (such as region), estimates at aggregate level may mask the need-based differentiations in local and national level requirements for reducing the use of UBAs [24, 25]. In order to reduce the number of UBA deliveries, knowledge of the factors related to the use of UBA deliveries at local and aggregate (national) level is required. Observing the limitations of a national level study in Bangladesh, Bhowmik et al. (2019) expressed the necessity for a spatial analysis of UBA delivery and the formulation of policy that reflects the needs of different geographic areas of the country [29].

The current study fills the aforementioned knowledge gaps including the meeting of the demand stressed by Bhowmik et al. (2019) by examining the variations in the level and predictors of UBA delivery across the geographic areas in Bangladesh, using spatial analysis techniques [29]. This study explores the question: what were the levels and determinants of UBA delivery in different geographic locations of Bangladesh in 2017/18, and which determinants were comparatively stronger in the regions with a high prevalence of UBA delivery? According to the literature, this study is the first to examine such spatial variations in the rate and determinants of UBA delivery in Bangladesh. The findings of this study will provide essential insights about UBA delivery by geographic location in Bangladesh. These insights will help to formulate specific interventions focused on the regions where the prevalence of UBA delivery is high, and allocating scarce resources on a priority basis.

## Methods

### Data

This study used the nationally representative data of the Bangladesh Demographic and Health Survey (BDHS) conducted in 2017/18 under the Demographic and Health Survey (DHS) Program. This survey covered all people in Bangladesh living in non-institutional residences. The BDHS was overseen by the National Institute of Population Research and Training (NIPORT) and ICF. In more than 90 countries, the DHS Program has provided assistance in conducting over 400 surveys, and the program has established a reputation in collecting and disseminating accurate, nationally-representative data in different disciplines of demography [33].

A two-stage stratified sampling technique was used by the 2017/18 BDHS. This survey selected 675 enumeration areas (EAs; which are known as clusters) in the first stage, using the probability proportional to the size of the cluster from 22 strata. The strata were created by demarcating each division (region) into urban city corporations, urban areas other than city corporations, and rural areas. The 2017/18 BDHS used the comprehensive list of EAs produced by the Bangladesh Bureau of Statistics, using the 2011 population census of Bangladesh. This census was the most recent census available before the 2017/18 BDHS. All the households in those selected clusters were listed at this stage. In the second stage of sampling, a total of 20,250 households were selected from the comprehensive household list of the specified clusters, using the systematic sampling technique. This technique allocated an average of 30 households to each cluster. Finally, 20,160 households were selected from 672 clusters, as three clusters were completely eroded by flood water. Of these selected households, 19,584 were occupied and 19,457 (99%) of these were successfully surveyed. A total of 20,127 women were finally interviewed from the 20,376 eligible 15–49 years old (reproductive age) ever-married women, giving a 99% response rate [15].

The 672 clusters were our unit of analysis. Characteristics of the clusters were created using the information of the women living in the respective clusters. These women were selected by applying the following criteria: i) had a minimum of one birth in the three years immediately prior to the 2017/18 survey, ii) answered the necessary questions related to birth attendants, and iii) responded to the questions associated with the candidate explanatory variables. A total of 5,011 (or 5,051 weighted) ever-married women of reproductive age (15–49 years) were found to meet these criteria. Out of the 40 candidate explanatory variables, 36 were created using the information of those 5,051 weighted women. In creating the remaining four candidate explanatory variables, some women were excluded because their responses to the relevant questions were missing. These four candidate explanatory variables were: women were informed that the pregnancy of the indexed birth had signs of complication, women’s participation in deciding on her own health care, women’s husbands’ education, and women whose indexed birth was caesarean, which, respectively, used 4,603 (or 4,646 weighted), 4,945 (or 4,987 weighted), 4,945 (or 4,987 weighted), and 5,006 (or 5,044 weighted) ever-married reproductive age women. In calculating values by cluster, associated weights (provided by the BDHS survey) were applied.

The geographic locations of the centres of the EAs were also collected by the 2017/18 BDHS. These geographic locations were used in the spatial analysis. In order to maintain the confidentiality of respondents’ own and community locations, the geographic locations of the clusters in urban areas were randomly displaced by up to two kilometres (km). Clusters in rural areas were randomly displaced by up to five kms, and an additional one percent of clusters that were selected randomly from rural areas were displaced by up to 10 kms. For these displacements, the selection of an appropriate buffer size is recommended so that an accurate idea about the area where the cluster is located is still possible [34].

Our study also used the Bangladesh Health Facility Survey (BHFS) data collected in 2017. The main objectives of the survey were to assess the availability, preparedness, and readiness of the health facilities. The survey initially selected 1,600 health facilities for investigation using a stratified random sampling technique. These 1,600 health facilities were selected from the entire range of registered facilities (19,811) in Bangladesh. This included all primary, secondary and tertiary level facilities in all eight divisions of the country, ensuring that this sample was nationally representative [35]. The BHFS collected information on inventory, care provision and geographic location of 1,524 health care facilities from the initial 1,600 facilities selected. Geographic locations and service providers’ information about these 1,524 facilities were used in this study [36].

### Variables

About 6.4% of the Bangladeshi women who gave birth at home in the three years preceding the 2017/18 BDHS used SBAs during their last delivery. It is interesting however, that 0.4% of women whose last births were at health facilities used UBAs and 46.7% of women whose last births in the three years preceding the survey were elsewhere (delivery hut and others) used UBAs (weighted calculation from 2017/18 BDHS raw data). These statistics indicate that the women receiving poor delivery assistance cannot be accurately identified by simply using the information on their places of delivery. Therefore, we decided to analyse the deliveries that actually used UBAs, rather than using the place of delivery and assuming UBA/SBA use.

The dependent variable for our study was the proportion of the selected women in the clusters whose last delivery in the past three years of the survey was assisted by a UBA. A UBA delivery refers to one that was assisted by a community health care provider, a trained and/or untrained traditional birth attendant, a non-government organization (NGO) worker, an unqualified doctor, a relative or friend, a neighbor, some other persons, or no one. An SBA delivery refers to one that was assisted by a qualified doctor, a family welfare visitor, a paramedic/midwife/nurse, a sub-assistant community medical officer or a community SBA [15].

To identify the best model, a total of 40 candidate explanatory variables were entered in an Exploratory Regression. The candidate explanatory variables were created using the independent variables analyzed in the existing literature for examining the determinants of the use of UBA/SBA. We classified each variable into as many categories as possible so that no category was omitted from the investigation. The complete list of the candidate explanatory variables is presented in S1 Table, which is available online (see Supporting information). The candidate explanatory variables were related to mothers’ current age and age at the indexed birth, the orders of the indexed births, women’s and their husbands’ education, work status of the women, mass media exposure, household’s wealth status, mothers’ healthcare decision-making, number of ANC contacts, delivery type of the indexed birth, whether or not the mothers had been informed about a pregnancy complication, experience of pregnancy termination, and the number of health facilities within 10 kilometres of the cluster that had at least one provider with delivery-related training. We included the presence of the health facilities within 10 kilometres for two reasons: i) a study in Zambia showed that the odds of providing emergency obstetric care to a cluster significantly reduced by 65% for every 10 kilometres increase in the distance between the cluster and the facility [37], and ii) selecting a 10 kilometres distance was expected to reduce the misclassification that could result from the displacement of the clusters and collecting information from the sampled health care facilities instead of the census of the health facilities [34].

### Descriptive analysis

**Fig 1:**
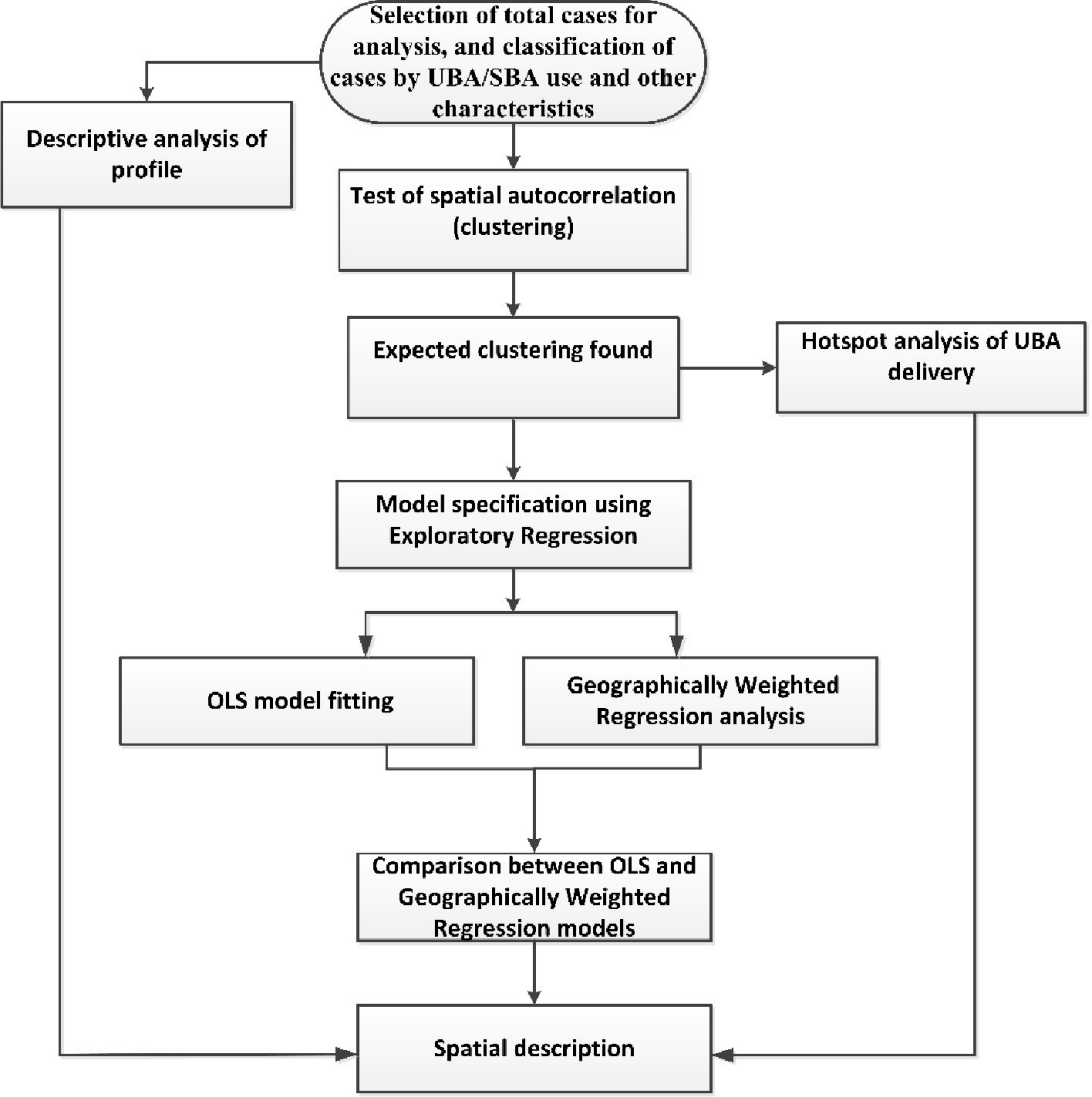
Flow chart of analysis. *UBA* = Unskilled Birth Attendant; *SBA* = Skilled Birth Attendant; *OLS* = Ordinary Least Squares.

The analysis process is presented in Fig 1. Characteristics of the clusters and respondents (women) were described using percentages and means. Differences in the level of UBA delivery by residence and region were examined using the chi-squared test developed by Pearson. The percentages and means relating to the respondent characteristics were calculated from the (women’s) individual sample weights, as provided by the survey. In the 2017/18 BDHS, the respondents were nested in the clusters and the clusters were nested in the strata. For this kind of nested data, a technique developed for complex survey design (accounting sampling strata, clusters and individual sample weights) is suggested for analysis. This technique is important for computing the weighted confidence interval and was used in this study to compute the percentages and means related to the individuals, and their confidence intervals. IBM SPSS Statistics for Windows, version 23.0 (IBM Corp., Armonk, N.Y., USA) was used to perform these analyses.

### Spatial analysis

The spatial autocorrelation analysis was performed using Global Moran’s I statistic for examining the pattern of spatial distribution of a value (clustered/dispersed/random) [38]. The Getis-Ord General G (General G) statistic was used to measure the level (high or low) of clustering. A larger or smaller z-score indicates that the clustering is stronger [38]. The UBA delivery rate was analyzed using the optimized hot spot analysis tool that identifies statistically significant hot spots and cold spots using the Getis-Ord Gi* (Gi*) statistic. A statistically significant hot spot is a cluster that has a high UBA delivery rate and is surrounded by other clusters with high rates, while a statistically significant cold spot is a cluster with a low UBA delivery rate surrounded by other clusters with low rates. The hot spot analysis identifies a high/low rate by comparing the local mean rate (the rates of a cluster and its neighboring clusters) with the global mean rate (the rates of all clusters). In this analysis, a z-score and a p-value are produced for each cluster, which identify the statistical significance of the differences between local and global means. Optimized hot spot analysis automatically corrects the results from the Gi* statistic for spatial dependence and multiple testing using the method of false discovery rate correction [38].

The observed spatial pattern of UBA delivery was then analyzed using a good fit Ordinary Least Squares (OLS) regression model to identify its predictors. Exploratory Regression was used to find a good fit OLS model. Exploratory Regression represents different models with different combinations of candidate explanatory variables and their goodness of fit. Like the Stepwise Regression, Exploratory Regression identifies the models with high Adjusted R-squared values. Unlike the Stepwise Regression, Exploratory Regression specifies a model that satisfies the assumptions of OLS regression model [38–40].

Results of an OLS regression model are reliable when the model satisfies all of the required assumptions of the method [38]. A properly specified OLS regression model should include the explanatory variables that are important for that model, which is reflected in the statistical significance of the coefficients of those variables. The model should have a reasonably high Adjusted R-squared value. In addition, there should not be redundancy (multicollinearity) among the explanatory variables, the residuals should be normally distributed, the model should be stationary, and the residuals should be free from spatial autocorrelation [39, 41]. Nevertheless, a regression model with statistically significant non-stationarity and spatially autocorrelated residuals is a suitable candidate for Geographically Weighted Regression analysis [38]. The variation in the strength (strong or weak) of an independent variable across the clusters is known as non-stationarity. This kind of variation across the clusters (non-stationarity) can be recognized using Geographically Weighted Regression analysis. Geographically Weighted Regression generates an equation for each cluster in contrast to the OLS fitting a single model to all data points in the whole study area [38]. Geographically Weighted Regression analysis through mapping the coefficients is also recommended if spatial autocorrelation of residual is evident in the OLS model [38]. If the Akaike’s Information Criterions (AICc) of Geographically Weighted Regression and OLS models differ by 3 or more, the model that produces a lower Akaike’s Information Criterion can be considered better [38].

The spatial analysis used clusters as the units of analysis. The proportions of the respondents in the clusters by specific characteristics, defined as the explanatory variables, indicated the characteristics of the clusters. These proportions were calculated by applying the respondents’ individual sample weights. The spatial analysis uses clusters as the units of analysis, and clusters do not furnish the necessary nesting and information required by the technique for complex sample design. Also, the spatial analysis tool does not have the option of using complex sample design. All the aforementioned spatial statistical analyses were performed using ArcGIS Desktop for Windows, version 10.5 (ESRI, Redlands, C.A., USA).

## Results

### Profile of clusters and respondents

Table 1 shows that deliveries of 47.1% (95% CI: 45.7–48.5) (weighted) of the selected women (respondents) took place in attendance of a UBA. The proportion of women who used a UBA in rural areas (52.5%) was much higher than that in urban areas (32.3%). The highest percentage of UBA deliveries (that is, the percentage of women with UBA delivery among the selected women in a category) was observed in the Sylhet region (59.6%), followed by Mymensingh (58.8%), Barishal (52.7%), Rangpur (50.9%), Chattogram (49.2%), Rajshahi (45.3%), Dhaka (39.7%) and Khulna (36.3%). The proportion of women with UBA delivery increased with the increase in their age, while that proportion declined with the increase in their education. The average weighted age of the respondents was 24.9 years, and women received 6.9 years of education on average.

**Table 1:** Characteristics of clusters, respondents and UBA deliveries.

Characteristics	Percentage of			
	Clusters^1^ (%)	Respondents (weighted %)	Weighted women with UBA delivery out of total women with UBA delivery in the country(95% CI)	Weighted women with UBA delivery out of total women in each category(95% CI)
**Residence**				**P-value = 0.000**
Urban	37.1	26.9	18.4 (16.2–20.8)	32.3 (28.8–36.0)
Rural	62.9	73.1	81.6 (79.2–83.8)	52.5 (49.6–55.4)
**Region**				**P-value = 0.000**
Barishal	10.6	5.7	6.4 (5.3–7.6)	52.7 (46.4–58.9)
Chattogram	13.7	21.2	22.1 (19.2–25.4)	49.2 (43.1–55.3)
Dhaka	15.3	25.6	21.6 (18.6–24.8)	39.7 (34.3–45.3)
Khulna	12.8	9.2	7.1 (5.9–8.5)	36.3 (31.4–41.4)
Mymensingh	11.5	8.5	10.7 (9.2–12.4)	58.8 (53.6–63.8)
Rajshahi	13.1	11.6	11.2 (9.4–13.3)	45.3 (40.0–50.7)
Rangpur	12.5	10.6	11.4 (9.4–13.8)	50.9 (43.5–58.2)
Sylhet	10.6	7.6	9.6 (7.9–11.6)	59.6 (52.3–66.5)
**Respondent’s age**				**P-value = 0.002**
15–24	96.7	53.1	50.6 (48.4–52.9)	44.9 (42.2–47.6)
25–34	95.2	41.0	42.9 (40.5–45.3)	49.2 (46.2–52.2)
35–49	34.8	5.9	6.5 (5.5–7.6)	52.2 (45.9–58.4)
**Respondent’s education**				**P-value = 0.000**
No education	28.6	6.3	9.5 (8.1–11.1)	70.9 (64.9–76.2)
Primary incomplete	62.1	17.4	25.1 (22.9–27.5)	68.0 (63.7–72.1)
Primary complete	47.9	10.2	13.5 (12.0–15.1)	62.3 (56.9–67.4)
Secondary incomplete	94.5	43.7	42.4 (39.9–45.0)	45.7 (43.0–48.4)
Secondary complete or higher	74.0	22.3	9.5 (8.2–10.9)	47.1 (44.7–49.5)
**Total**	-	100.0	100.0	47.1 (44.7–49.5)
**Respondent’s mean age and education**				
Respondent’s weighted mean age (95% CI)			24.9 (24.7–25.0)	
Respondent’s weighted mean years of education (95% CI)			6.9 (6.7–7.1)	

Total number of clusters = 672; Total weighted number of respondents = 5051;

^1^The percentages of clusters by age and education do not sum up to 100 because the same categories of age and education exist in multiple clusters.

Source: BDHS, 2017/18.

Among the total number of women in Bangladesh who experienced UBA delivery, 81.6% resided in rural areas. Contributions of the Chattogram region (22.1) and Dhaka region (21.6) to the total number of women with UBA delivery in the country were remarkably higher than that in other regions, while Barisal’s contribution (6.4%) to that number was lowest.

Out of the total clusters, 62.9% were from rural areas (Table 1). The highest proportion of clusters (15.3%) were from the Dhaka region. Among the selected women, 73.1% were from rural areas. More than a quarter (25.6%) of the respondents were from the Dhaka region.

### Spatial autocorrelation of UBA delivery

The Global Moran’s Index (0.191440) and z-score (12.612290) indicated a statistically significant (p *<* 0.001) clustering of UBA delivery in Bangladesh. A high clustering was reflected in Getis-Ord General G statistic (z-score = -3.994664, p-value *<* 0.001).

### Hot spot analysis of UBA delivery

**Fig 2:**
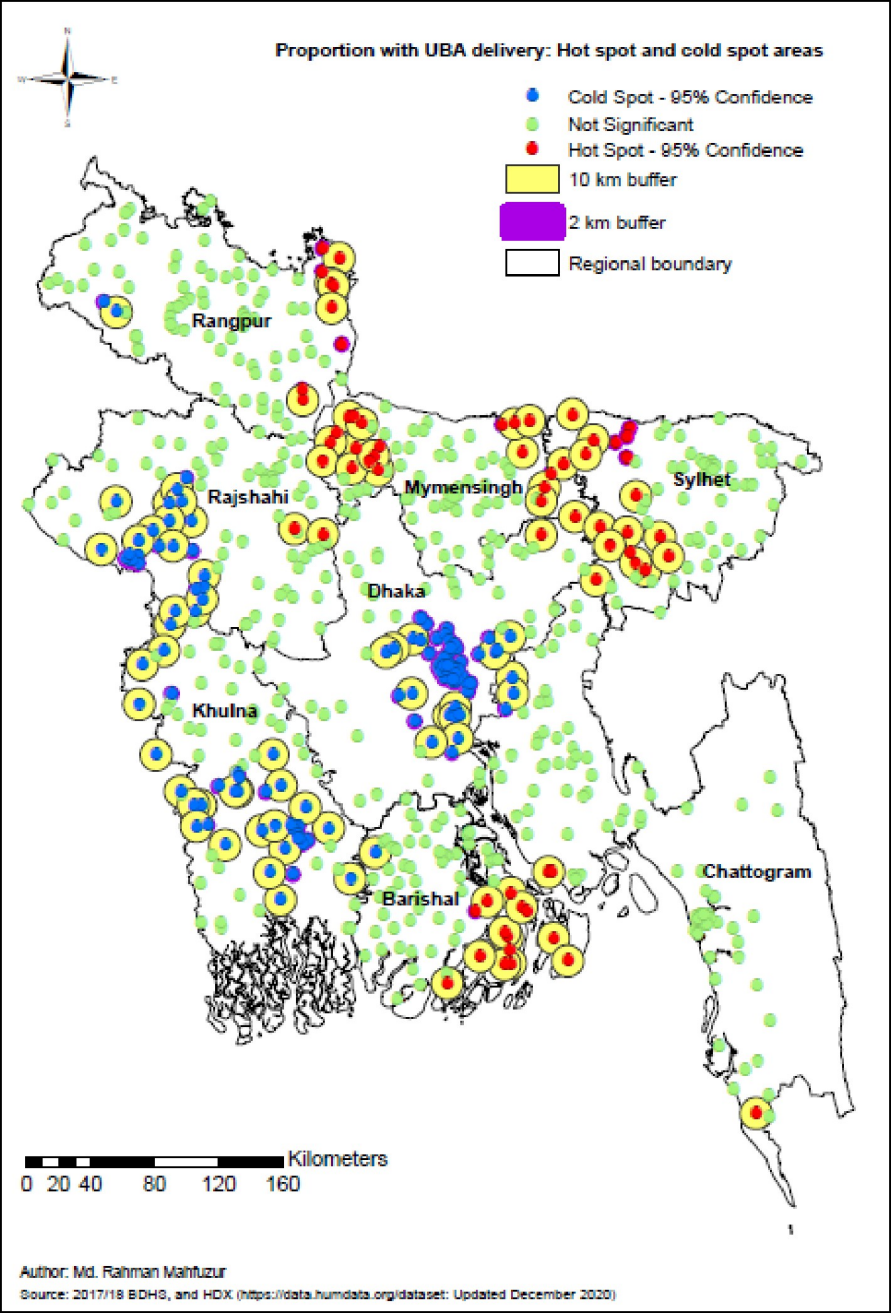
Optimized hot spots and cold spots of UBA delivery in Bangladesh, BDHS 2017/2018.

Fig 2 shows the hot and cold spots (clusters) that were statistically significant. As the clusters were randomly displaced (see data section), buffers of 10 kilometers and two kilometers were created, respectively, around rural and urban significant clusters. The results revealed a total of 67 statistically significant hot spots in Bangladesh. The eastern and northwestern areas of the Mymensingh region together comprised the highest percentage of the total hot spots (29.9%), followed by the Sylhet region (northwestern and western areas comprised 26.9%), the southeastern area of Barishal region (19.4%), the northeastern area of Rangpur region (13.4%), the Chattogram region (southern and western parts comprised 6%), the eastern area of Rajshahi region (3%), and the northeastern area of the Dhaka region (1.5%). The Khulna region had no statistically significant hot spot of UBA delivery. Out of the 139 cold spots, 48.2%, 30.2% and 17.3% were in Dhaka, Khulna and Rajshahi, respectively.

### Results of OLS regression, and comparison between OLS and GWR diagnostics

The results of a good fit OLS model are presented in Table 2. This model was specified with the help of Exploratory Regression analysis. The robust and usual p-values indicated the significance of the coefficients (<0.01) of the explanatory variables (Table 2).

**Table 2:** Summary of OLS results for UBA delivery in Bangladesh, BDHS 2017/18.

Variable	Coefficient	SE	Robust SE	P	Robust P	VIF
Intercept	0.40	0.039	0.046	< 0.01	< 0.01	-
Women with indexed birth-order 2^+^	0.12	0.039	0.045	< 0.01	< 0.01	1.10
Women with no or primary incomplete education	0.19	0.043	0.047	< 0.01	< 0.01	1.45
Women with secondary complete or higher education	-0.23	0.041	0.040	< 0.01	< 0.01	1.53
Women from poorest household wealth status	0.32	0.034	0.036	< 0.01	< 0.01	1.31
Women from poorer household wealth status	0.25	0.038	0.045	< 0.01	< 0.01	1.09
Women had 4^+^ ANC during the pregnancy of indexed birth	-0.26	0.031	0.035	< 0.01	< 0.01	1.28

All variables are weighted proportions in clusters; VIF: Variance Inflation Factor; SE: Standard Error.

Table 3 represents the results of the diagnostic tests. Table 3 shows that the selected model explained about 51% of the variability in UBA delivery (Adjusted R^2^ = 0.51), which is higher than the usual minimum acceptable level of R-squared (0.5) for the OLS model. The joint Wald-Statistic demonstrated an overall significance of the model (p < 0.001), and there was no evidence of redundancy (multicollinearity), reflected in the low value of variance inflation factor (< 7.5). The non-significant Jarque-Bera statistic (p > 0.10) suggests that the residuals of the model were distributed normally (i.e., the model was unbiased). A statistically significant Koenker (BP) statistic (p < 0.001) reveals that the modelled relationship may vary over geographic and data spaces, that is, the model is non-stationary. The residuals were found to be spatially autocorrelated, reflected in a statistically significant residual spatial autocorrelation (Moran’s I). The significant non-stationarity of the model and residual spatial autocorrelation rendered the selected explanatory variables suitable for Geographically Weighted Regression analysis [38]. Therefore, we conducted a Geographically Weighted Regression analysis to improve the (OLS) model.

**Table 3:** Summary of diagnostic test results of OLS and Geographically Weighted Regression for UBA delivery in Bangladesh, BDHS 2017/18.

Diagnostic tests		Values of diagnostic tests	
		OLS	Geographically Weighted Regression
Number of observations (clusters)	:	672	672	
Multiple R-squared	:	0.514	0.534
Adjusted R-squared	:	0.510	0.517
Joint F-statistic	:	117.24 (P < 0.001)	-
Joint Wald statistic	:	1048.11 (P < 0.001)	-
Jarque-Bera Statistic	:	4.56 (P = 0.102)	-
Koenker (BP) Statistic	:	26.27 (P < 0.001)	-
Residual spatial autocorrelation (Moran’s I)	:	0.05 (P < 0.01)	-
Overall model VIF	:	1.53	-
Akaike’s Information Criterion (AICc)	:	-244.58	-249.66
Residual squares	:	-	25.58
Effective number	:	-	24.98
Sigma	:	-	0.199

VIF: Variance Inflation Factor.

Overall, the Geographically Weighted Regression analysis in our study improved the model estimates, compared to that obtained using the OLS. The Adjusted R-squared value increased to 0.52 in the Geographically Weighted Regression from 0.51 in the OLS (see Table 3). The Akaike’s Information Criterion (AICc) value of the Geographically Weighted Regression (-249.66) was 5.08 points lower than that of the OLS (-244.58), which indicates that the Geographically Weighted Regression model is better than the OLS model [38].

### Results of Geographically Weighted Regression (GWR)

**Fig 3:**
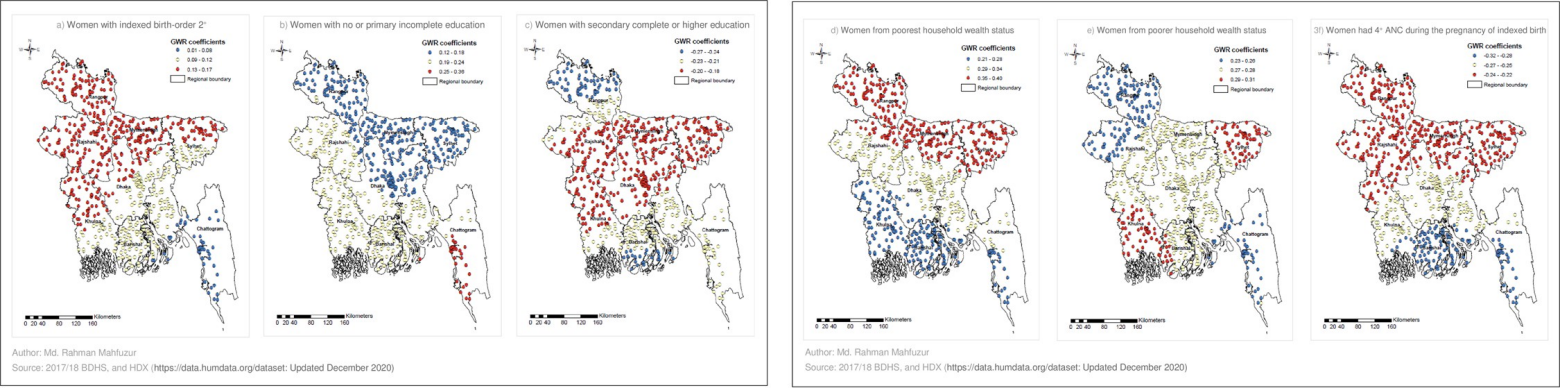
Geographically Weighted Regression coefficients of selected variables for predicting UBA delivery in Bangladesh, BDHS 2017/18.

The strengths of predicting UBA delivery by the explanatory variables in different geographic areas are presented in Fig 3 (Fig 3A–3F). Poorest household wealth status (coefficient range: 0.35–0.28), poorer household wealth status (coefficient range: 0.29–0.26), and women with no or some primary education (coefficient range: 0.25–0.18) had larger coefficients compared to other predictors.

An explanatory variable in a cluster was considered a strong predictor of UBA delivery if the coefficient of that variable for that cluster was in the largest range, which is indicated in red in Fig 3. As shown in Fig 3A, the variable “Women whose indexed birth order was two or more” exerted a strong positive influence on UBA delivery in the regions of Mymensingh, Rajshahi and Rangpur, and in the northern parts of Dhaka, Khulna and Sylhet. The variable “women’s some primary education” only appeared as a powerful predictor of delivery by UBA in the western part of the Chattogram region (Fig 3B). The variable “women’s secondary complete (all secondary) or higher education” showed a strong negative influence on UBA delivery in the regions of Dhaka, Mymensingh, Rajshahi and Sylhet (Fig 3C).

The variable “Women from poorest household wealth status” exerted a strong positive influence on UBA delivery in the regions of Mymensingh, Rangpur and Sylhet, and in northeastern Dhaka and northern Rajshahi (Fig 3D). Fig 3E shows that being a member of a household with poorer wealth status was a positive and strong predictor of UBA delivery in Sylhet, Khulna, and in the western part of Barishal. Receiving four or more ANC exerted a strong negative influence on UBA delivery in Mymensingh, Rajshahi, Rangpur, Sylhet, and in the northern parts of Dhaka and Chattogram.

## Discussion

This study aimed to reveal the variations in the level and predictors of UBA delivery over the geographic areas in Bangladesh, using advanced spatial statistical techniques. To the best of our knowledge, this is the first article revealing that the strength of the predictors of UBA delivery varied over the geographic areas in Bangladesh. Only three regions (Mymensingh, Sylhet and Barishal) accounted for more than three quarters of the total UBA delivery hot spots. Our findings showed that the order of the birth in question (indexed birth) two or higher, no or some primary education, poorest and poorer household wealth status, and having less than four ANC visits were the key predictors of higher UBA delivery rates, while all secondary education or higher was the key predictor of lower UBA delivery rates. Geographically Weighted Regression analysis showed that the factors that exerted a strong influence on UBA delivery in the regions of Bangladesh were different. As the model-building and the analyses in this study were done using recognized statistical techniques, results of this study can be used for prioritizing strategic action by geographic area for reducing the rates of UBA delivery in Bangladesh.

The significant UBA delivery hot spots were mainly noticed in the eastern and northwestern areas of Mymensingh, the northwestern and western areas of Sylhet, the southeastern area of Barishal and the northeastern area of Rangpur. Khulna was the safest region containing no significant hot spot. About 96% of the cold spots were located in Dhaka, Khulna and Rajshahi regions. Other studies also indicated a significant spatial variation in UBA delivery across Bangladeshi regions [29, 42]. This variation may be because of the differences in the sociocultural and socioeconomic characteristics of women in different regions. The practice of “Purdah” (concealing women in public) in the Sylhet region is one of the key barriers to changing women’s mobility and overall lifestyle [43]. The northeastern area of Rangpur also faces difficulties as it is affected by seasonal poverty and hunger [44]. Besides these, other socioeconomic factors that could be associated with the spatial variation in UBA delivery are discussed in later sections.

Although the study of Teshale et al. (2020) in Ethiopia found that, along with the other factors, perceiving the distance from health facility as a big problem was a strong predictor of UBA delivery, analysis of the proximity of health facility with a trained provider in our study found that proximity as a nonsignificant predictor of UBA delivery [30]. The stall in MMR in some middle-income countries (including Bangladesh), despite the increase in the coverage of maternal health services, indicates that there are factors other than service coverage that are important in improving health and the uptake of health services [4]. We found that about 81% of our study clusters had at least one facility with at least one provider with delivery-related training within 10 kilometres of the clusters (calculated from raw data). It should be noted that women in the southern area of the Barisal region may face extra challenges in accessing health services as this region is a delta island where transportation is poor and waterways are the main medium of transportation [45]. Although 81% of the clusters had access to a healthcare facility, not all facilities were equally equipped with trained providers. The percentages of facilities with staff trained in delivery care in Mymensingh (35%), Sylhet (38%), Barishal (34%) and Rangpur (35%) are much lower than that in the Khulna (42%), Chattogram (53%) and Dhaka (55%) regions [36].

In this study, there was a positive and strong association between the variable “Second or higher order delivery (birth order/parity)” and the rate of UBA delivery in the Mymensingh, northern Sylhet and Rangpur regions. These regions had a higher prevalence of UBA delivery hot spots. Second or higher order delivery also manifested a positive and strong relation with UBA delivery in the northern Khulna (the safest region). This finding of positive association is analogous to that of other research on UBA delivery in Bangladesh and elsewhere [27, 30, 42]. This finding may be explained by women feeling more experienced in their second or higher order deliveries, and thus opting for free or cheap UBAs for assistance [46].

As in other studies, no or some primary education showed a positive association with UBA delivery, and all secondary or higher education showed a negative association with UBA delivery [27, 29, 30]. No or some primary education appeared as a powerful predictor of deliveries by UBA in the southwestern areas of Chattogram, while all secondary or higher education appeared as a key predictor of lower rates of UBA delivery in the hot spot-stricken regions of Mymensingh and Sylhet, and in the safest region–Khulna. Women who have secondary or higher education are usually well aware of the complications related to their health and the use of traditional or unscientific cheaper treatment, which in turn influences them to consider using the medical services available [29, 47].

This study found that the poorest household wealth status was positively and strongly associated with delivery by UBA in the high UBA hot spot prevalent regions of Mymensingh, Sylhet and Rangpur. While the poorer household wealth status appeared to be a positive and strong predictor of UBA delivery in Sylhet and some parts of Barishal. Studies in different countries also found a higher odds of having UBA delivery among mothers from poorer households compared to that among those from richer households. It may be difficult for the women from poorer households to bear the high monetary cost of SBA services, as these costs can include the cost of the service itself, travel costs and ongoing financial stress caused by the comparatively higher expense of SBA [20, 29, 42, 48].

When exploring the association between UBA use and the number of ANC visits experienced, we found a negative relationship between UBA delivery and receiving four or more ANC visits. For a positive pregnancy experience, the focused ANC model set out by the World Health Organization suggests that women receive at least four ANC visits, while the standard ANC model suggests that they receive at least eight ANC visits [49]. As the average number of ANC visits received by the women in this study was below four (3.8; weighted mean calculated from 2017/18 BDHS data), we analyzed the proportion of women who had received at least four ANC visits. A moderate percentage of women (47%) in Bangladesh had at least four ANC contacts [15]. The UBA delivery hot spot-affected regions where the variable of “Four or more ANC visits” exerted a strong negative influence on UBA delivery were the entire regions of Mymensingh, Sylhet and Rangpur. Other studies also found that those who attended at least four sessions of ANC had much lower odds of using UBAs for their deliveries [50, 51]. The reason for this is that an increased number of ANC visits provide women with more information about the pregnancy and can assess delivery-related risks at different stages of pregnancy that may require SBA assistance [50].

The key strength of this study over other studies is that it used a robust method for specifying a model by using a wide variety of variables with a large sample size from the most recent nationally representative population-based survey. In addition to identifying the predictors of UBA delivery at a national level, this study revealed the prevalence and predictors of UBA delivery at the local level.

This study also has some limitations. The BDHS randomly displaced the GPS locations of clusters (see data section). Although these displacements may affect the estimates of cluster effects, the randomness of the displacement may adjust that estimate-error to a large extent, however the locations of hot spots and cold spots (clusters) cannot be identified accurately because of the displacements. As per the recommendation of Burgert and Prosnitz, this problem was minimized substantially by using 2 km and 10 km buffers, respectively, around urban and rural significant hot spots and cold spots [34]. These buffers are expected to give an accurate assessment about the area where those spots were located.

In calculating the proportions of women with some characteristics, some cases were excluded due to missing values, however, none of the variables with excluded cases was specified by the Exploratory Regression in our final models (OLS and GWR). As this study was based on mothers’ self-reports, there may be some recall bias. It should also be noted that, although this study did not find that the proximity of women to health facilities with trained delivery care providers was a strong predictor of using SBAs, this may be due to the healthcare facility misclassification and/or the high density of healthcare facilities. As the healthcare facility survey did not collect information from all the health facilities in the country and the clusters were randomly displaced, a health facility could be misclassified as closer to a cluster than it actually was. The impact of this misclassification has been minimized by linking clusters that have desired health facilities within 10 kilometres, thus eliminating the misclassification bias to a large extent. Although we relied on a survey for the healthcare facility data, our back calculation showed that about 81% clusters had at least one facility with at least one provider with delivery-related training within a 10-kilometre radius, which indicates significant coverage. Our findings that this high level of service provision did not significantly impact on the use of SBAs in all regions aligns with the findings of the BMMS report. This study also argues that increasing the provision of maternal healthcare services in middle-income countries, such as Bangladesh, may not result in an improvement in maternal health and increase the use of health facilities [4]. We argue that a number of other factors also influence women’s choices around delivery care.

Our review of the literature found that other studies on UBA use did not include a variable related to the proximity of healthcare facilities in their spatial analysis. In our study, we included this variable in the Exploratory Regression analysis, but did not specify that variable in our final model, and none of the analyses that furnish the main part of the results of our study included the variable related to the proximity of healthcare facilities. Finally, cause-effect relationships cannot be inferred from this study due to its cross-sectional nature. We suggest that an independent project should examine the effects of the locally-relevant strong predictors for use of SBAs, through the collection and analysis of longitudinal data.

## Conclusions

The current study revealed a spatial variation of UBA delivery across the regions of Bangladesh. The hot spots of UBA delivery were mainly found in the Mymensingh, Sylhet, Barishal, and Rangpur regions. The strengths of the predictors found to be significant at the national level varied across the regions. As multiparous women in some regions were found to have higher rates of UBA use, we recommend that health and family planning services should focus on the multiparous women in the Mymensingh, Sylhet and Rangpur regions. Improving the socioeconomic conditions of women in all the hot spot-stricken regions may increase the use of SBAs and the enhancement of women’s education, to at least secondary education level, may increase the rate of use of SBAs in the Mymensingh and Sylhet regions. Encouraging women to attend at least four ANC contacts is expected to increase the utilization of SBAs in the Mymensingh, Sylhet and Rangpur regions.

As enhancement of education and socioeconomic conditions is a long-term process, alternative interventions could be used to achieve the UN goals regarding the use of SBAs. The steps recommended in this study can be applied to the whole country, but prioritizing the rollout of these interventions in the hot spot-stricken areas is expected to drive a fast increase in SBA use. The recommended interventions include the following; UBA delivery hot spots should immediately be set as priority areas for visits by health and family planning field workers (such visits currently exist in Bangladesh) [15]; along with the delivery of the existing services at the doorsteps of the women living in the hot spots, the women should be informed about the risks related to delivery and the advantages of ANC visits and using SBAs; ANC services should be introduced in the existing community clinics at a very low cost or free, especially in the hot spot-stricken areas; at least one trained delivery care provider should be employed in the nearest health facility to each cluster, especially in those which were identified as hot spots. The introduction of low cost or free ANC and child delivery services in the target areas will lower the poverty-related barriers that prevent the use of modern and expensive services.

## Implications for policy planners and programs

This paper includes maps of UBA delivery hot spots and their associated factors that will be very helpful in formulating and implementing precision public health policies and programs. For example, the hot spot map provides information on where to target interventions aimed at alleviating maternity-related morbidity and mortality. This information will also be very helpful when allocating resources to the areas of the health sector tasked with reducing the rate of UBA delivery. This mapped evidence can also be used in monitoring the impacts of health and family planning programs across the regions. In addition, these findings will help policy makers to rapidly formulate specific strategies at both the local and national levels. It is hoped that this study will inform the necessary actions required by the government and other agencies to improve many aspects of maternal health in Bangladesh, in both a rapid and cost-effective manner.

## Supporting information

S1 Table Candidate explanatory variables included in the Exploratory Regression tool. (DOCX)
