# Supplementary material for: Exploring spatial variations in level and predictors of unskilled birth attendant delivery in Bangladesh using spatial analysis techniques: Findings from nationally representative survey data
Source: PLoS One. 2022 Oct 25;17(10):e0275951. doi: 10.1371/journal.pone.0275951 (PMC9595510; doi:10.1371/journal.pone.0275951)
Supplement: S1 Table — (DOCX) [file pone.0275951.s001.docx]

**S1 Table. Candidate explanatory variables included in the Exploratory Regression tool.**

| **Broader category** | **Specific variables** |
| --- | --- |
| Mother’s current age | 1) Weighted proportion of women aged 15-24 years in cluster  2) Weighted proportion of women aged 25-34 years in cluster  3) Weighted proportion of women aged 35-49 years in cluster |
| Mother’s age at birth | 4) Weighted proportion of women who were 18 or older at the time of indexed birth in cluster |
| Order of birth | 5) Weighted proportion of women whose indexed birth’s order was second or higher in cluster |
| Mother’s education | 6) Weighted proportion of women with no education in cluster  7) Weighted proportion of women with primary incomplete education in cluster  8) Weighted proportion of women with primary complete education in cluster  9) Weighted proportion of women with secondary incomplete education in cluster  10) Weighted proportion of women with secondary complete education in cluster  11) Weighted proportion of women with higher education in cluster  12) Weighted proportion of women with no or primary incomplete education in cluster  13) Weighted proportion of women with secondary complete or higher education in cluster |
| Husband’s education | 14) Weighted proportion of women whose husbands had no education in cluster  15) Weighted proportion of women whose husbands had primary incomplete education in cluster  16) Weighted proportion of women whose husbands had primary complete education in cluster  17) Weighted proportion of women whose husbands had secondary incomplete education in cluster  18) Weighted proportion of women whose husbands had secondary complete education in cluster  19) Weighted proportion of women whose husbands had higher education in cluster |
| Mother’s work status | 20) Weighted proportion of women who were not currently working in cluster |
| Exposure to mass media | 21) Weighted proportion of women who read newspaper/magazine at least once in a week in cluster |

Continued **Table S1**

|  | 22) Weighted proportion of women who listened to radio at least once in a week in cluster  23) Weighted proportion of women who watched TV at least once in a week in cluster  24) Weighted proportion of women who read newspaper/magazine or listened to radio or watched TV at least once in a week in cluster |
| --- | --- |
| Household wealth status | 25) Weighted proportion of women from poorest household wealth status in cluster  26) Weighted proportion of women from poorer household wealth status in cluster  27) Weighted proportion of women from middle household wealth status in cluster  28) Weighted proportion of women from richer household wealth status in cluster  29) Weighted proportion of women from richest household wealth status in cluster  30) Weighted proportion of women from poorest or poorer household wealth status in cluster |
| Health care decision making | 31) Weighted proportion of women whose health care is decided by themselves alone in cluster  32) Weighted proportion of women whose health care is decided by themselves and their husbands in cluster  33) Weighted proportion of women whose health care is decided by their husbands alone in cluster  34) Weighted proportion of women whose health care is decided by themselves alone, or themselves and their husbands in cluster |
| Antenatal care (ANC) visit | 35) Weighted proportion of women who had no ANC visit during the pregnancy of the birth in question (indexed birth) in cluster  36) Weighted proportion of women who had four or more ANC visits during the pregnancy of the birth in question (indexed birth) in cluster |
| Delivery type | 37) Weighted proportion of women whose birth in question was caesarian in cluster |
| Pregnancy complication | 38) Weighted proportion of women who were informed that the pregnancy of indexed birth had complication signs in cluster |
| Pregnancy termination | 39) Weighted proportion of women who never had terminated pregnancy (miscarriage/abortion/still birth) in cluster |
| Health facility | 40) Number of health facilities in 10 kilometers of cluster having at least one provider with delivery care related training |
